# Cytotoxic and genotoxic effects of oxime β-lapachone in human cancer cells: selectivity toward NCI-H460 and insights from molecular docking

**DOI:** 10.1007/s13577-026-01427-8

**Published:** 2026-07-27

**Authors:** Maria Francilene Souza Silva, Lara Polyana Silva Ramos, Fátima de Cássia Evangelista de Oliveira, Bruno Marques Soares, Daniel Pascoalino Pinheiro, Igor Frederico da Silveira Ramos, Rayran Walter Sousa, Victória Laysna dos Anjos Santos, Arlan de Assis Gonsalves, Paulo Michel Pinheiro Ferreira, Heurison Sousa e Silva, Cleônia Roberta de Melo Araújo, Claudia Pessoa, Marcia dos Santos Rizzo, Marcília Pinheiro Costa

**Affiliations:** 1 Laboratory of Experimental Oncology, Research and Development Center for Pharmaceuticals (NPDM), Federal University of Ceará, Fortaleza, CE 6430-275 Brazil; 2 Interdisciplinary Laboratory for Advanced Materials (LIMAV), Graduate Program in Pharmaceutical Sciences, Federal University of Piauí, Teresina, PI 64049-550 Brazil; 3 Laboratory of Experimental Cancerology (LabCancer), Department of Biophysics and Physiology, Federal University of Piauí, Teresina, PI 64049-550 Brazil; 4 Graduate Program in Health and Biological Sciences, Federal University of the São Francisco Valley, Petrolina, PE 56304-917 Brazil; 5 Department of Physics, Federal University of Piauí, Teresina, PI 64049-550 Brazil; 6 Health Sciences Center, Center for Experimental Biology (Nubex), University of Fortaleza (UNIFOR), Fortaleza, CE Brazil

**Keywords:** Naphthoquinone, Oximation, Apoptosis, DNA damage, Non-small cell lung cancer

## Abstract

β-Lapachone exhibits potent anticancer activity although its clinical application remains limited by toxicity and mechanisms of resistance. Therefore, structural modifications have been explored to improve its pharmacological profile. This study evaluated the cytotoxic, genotoxic, and toxicological effects of the oxime derivative β-lapachone oxime (Oxβ-Lp), together with its predicted pharmacokinetic properties and potential molecular interactions. Oxβ-Lp displayed cytotoxic activity against all tested cancer cell lines (NCI-H460, PC9, K562, and HepG2) after 72 h of exposure, with the greatest potency and selectivity observed in NCI-H460 non-small cell lung cancer cells (IC₅₀ = 1.88 µM; SI = 13.1). Mechanistic analyses demonstrated reduced cell viability, mitochondrial membrane depolarization, DNA damage, and induction of apoptosis, without significant cell cycle arrest. In *Allium cepa*, Oxβ-Lp did not alter the mitotic index or induce micronucleus formation but promoted chromosomal aberrations and DNA strand breaks. The *Artemia salina* assay indicated high acute toxicity (LC₅₀ = 16.80 µg/mL). Molecular docking suggested a potential interaction between Oxβ-Lp and NQO1, with binding energies comparable to those of dicoumarol and similar interaction patterns within the catalytic site. Overall, these findings demonstrate that Oxβ-Lp exhibits selective cytotoxicity against NCI-H460 cells and promotes apoptosis associated with mitochondrial dysfunction and DNA damage. Although the molecular mechanisms underlying its biological activity require further investigation, Oxβ-Lp represents a promising scaffold for developing novel anticancer agents.

**Supplementary Information:**

The online version contains supplementary material available at 10.1007/s13577-026-01427-8.

## Introduction

Several strategies involving the modification and hybridization of prototype anticancer agents have been proposed to improve their therapeutic efficacy and pharmacological profile. These approaches aim to overcome common limitations associated with many anticancer drugs, including high lipophilicity, poor bioavailability, and significant toxicity [1–3]. In the search for novel anticancer agents, quinones have attracted considerable attention owing to their unique chemical structure and broad potential for structural modification [4].

Quinone-class drugs are notable for their potent anticancer activity and well-established clinical applications. Doxorubicin, daunorubicin, and mitomycin C are representative examples that have been extensively used in cancer therapy. Consequently, numerous studies have focused on the development of modified quinones to address limitations, such as poor solubility, chemical instability, multidrug resistance, lack of selectivity, adverse side effects, and systemic toxicity [5, 6]. In addition, many efforts involving chemical and structural modifications of natural naphthoquinones isolated from *Handroanthus impetiginosus*, including lapachol, α-lapachone, and β-lapachone, have led to the development of novel compounds with improved chemical, physicochemical, and pharmacological properties [7, 8].

β-Lapachone exhibits potent anticancer activity and has demonstrated greater efficacy in several tumor types reported to present elevated endogenous levels of NAD(P)H:quinone oxidoreductase 1 (NQO1), including breast, non-small cell lung, pancreatic, colon, and prostate cancers [9]. NQO1 has been recognized as an important mediator of β-lapachone bioactivation, promoting redox cycling, excessive reactive oxygen species (ROS) generation, DNA damage, and ultimately cancer cell death [10]. Consequently, cancer cells exhibiting high endogenous NQO1 expression are particularly susceptible to β-lapachone-mediated cytotoxicity. Despite its promising antitumor activity, clinical studies have reported adverse effects associated with β-lapachone administration, including nausea and vomiting. These limitations have stimulated the development of structural derivatives aimed at improving efficacy while reducing toxicity toward non-tumor cells [8].

In addition to its well-established NQO1-dependent bio-activation mechanism, β-lapachone has been reported to exert anti-proliferative and anti-metastatic effects through modulation of multiple intracellular signaling pathways, including Akt/mTOR inactivation, oxidative stress induction, DNA damage, and apoptosis activation in different tumor models [11, 12]. These findings reinforce the relevance of quinone-based derivatives as promising scaffolds for the development of multi-target anticancer agents.

An important strategy in anticancer drug discovery is the development of multifunctional molecules capable of simultaneously modulating distinct cellular targets. In this context, oximation has emerged as a valuable approach for generating compounds with enhanced pharmacological properties and broader biological activities [13, 14]. In this context, Araújo et al. [15] synthesized oximize derivatives of lapachol and β-lapachone, to improve their pharmacological properties. One of these compounds, 2H-naphtho[1,2-b]pyran-5,6-dione, 3,4-dihydro-2,2-dimethyl-6-oxime (oxime β-lapachone, Oxβ-Lp), is a naphthoquinone-derived oxime synthesized from lapachol (2-hydroxy-3-(3-methyl-2-butenyl)−1,4-naphthalenedione). This derivative demonstrated significant anticancer activity against several cancer cell lines, including SF-295 (central nervous system), HCT-116 (colon), NCI-1975 (lung), HL-60 (leukemia), and L929 (murine fibroblast), with IC_50_ values of 3.47, 7.00, 21.57, 3.84 and 24.63 µM, respectively.

Given its promising anticancer activity, further investigation of Oxβ-Lp is warranted. Therefore, the present study aimed to evaluate the cytotoxic activity of Oxβ-Lp in additional human cancer cell lines, assess its genotoxic effects, and investigate cellular events associated with its cytotoxic activity in NCI-H460 non-small cell lung cancer cells. Furthermore, the genotoxic potential of Oxβ-Lp was evaluated using *Allium cepa* cells, while its acute toxicity was assessed using an *Artemia salina* model. In addition, the absorption, distribution, metabolism, excretion, and toxicity (ADMET) properties of Oxβ-Lp were predicted. Because NQO1 is an important enzyme involved in the bio-activation of β-lapachone and related quinones, molecular docking studies were performed as an exploratory approach to assess the potential interaction of Oxβ-Lp with this enzyme. However, the present study was not designed to establish a causal relationship between NQO1 activity and Oxβ-Lp sensitivity, and any association should be interpreted with caution and considered hypothesis-generating.

## Materials and methods

### Materials

**Fig. 1:**
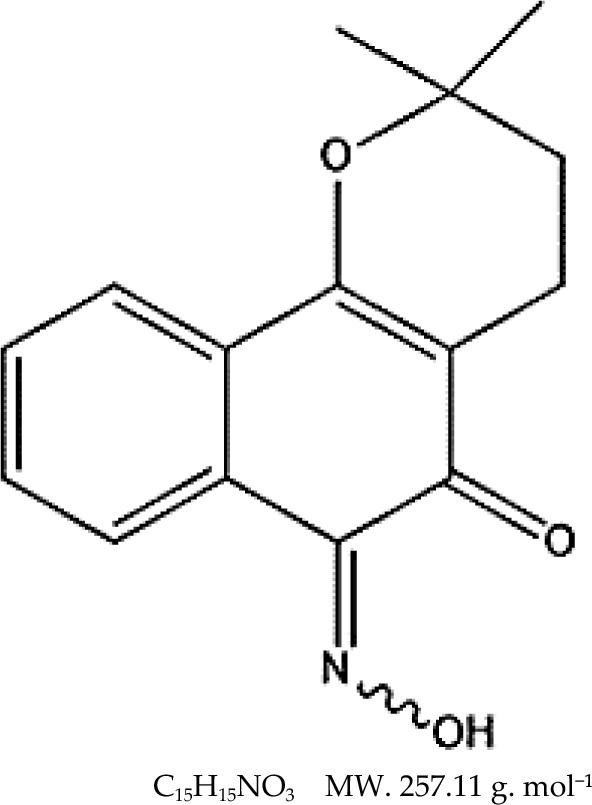
Chemical structure of the β-lapachone oxime (Oxβ-Lp)

Oxime β-lapachone (Oxβ-Lp, 2-hydroxy-3-(3-methyl-2-butenyl)−1,4-naphthalenedione oxime: purity > 99%, Fig. 1) was synthesized as described by Araújo et al. [15]. MTT [3-(4,5-dimethylthiazol-2-yl)−2,5-diphenyltetrazolium bromide], propidium iodide, rhodamine 123, doxorubicin, and cyclophosphamide were purchased from Sigma-Aldrich Co. (St. Louis, MO, USA). RPMI-1640 medium, Dulbecco's modified Eagle medium (DMEM), fetal bovine serum (FBS), penicillin, and streptomycin were purchased from Gibco™ (Invitrogen, Carlsbad, CA). The Panoptic staining kit was purchased from LaborClin (Rio de Janeiro, RJ, BR). All other reagents and chemicals used in this study were of analytical grade and were used according to the manufacturers’ instructions.

### Cell lines and cell cultures

The human cancer cell lines K562 (chronic myeloid leukemia), HepG2 (hepatocellular carcinoma), NCI-H460 (non-small cell lung cancer), and PC9 (non-small cell lung cancer) were obtained from the National Cancer Institute (Bethesda, MD, USA). The non-tumorigenic cell line NCTC clone 929 (L929; mouse fibroblast) was obtained from the Rio de Janeiro Cell Bank (Rio de Janeiro, RJ, Brazil).

Cells were cultured in RPMI-1640 or Dulbecco’s modified Eagle medium (DMEM) supplemented with 10% fetal bovine serum (FBS), 100 U/mL penicillin, and 100 μg/mL streptomycin. Cultures were maintained at 37 °C in a humidified atmosphere containing 5% CO₂.

### Cytotoxicity against cell lines

Cell viability was evaluated using the MTT reduction assay, based on the conversion of the yellow tetrazolium salt 3-(4,5-dimethylthiazol-2-yl)−2,5-diphenyltetrazolium bromide (MTT) into insoluble purple formazan crystals by metabolically active cells [16].

Briefly, the cells were seeded into 96-well plates containing 100 μL of culture medium at densities of 3 × 10^5^ cells/well (K562), 1 × 10^5^ cells/well (HepG2), 7 × 10^4^ cells/well (NCI-H460), 1 × 10^5^ cells/well (PC9), and 5 × 10^3^ cells/well (L929). Oxβ-Lp, dissolved in 1% dimethyl sulfoxide (DMSO), was tested at concentrations ranging from 0.05 to 100 μM. Doxorubicin (0.1 μM) was used as a positive control, whereas control cells received an equivalent concentration of DMSO.

After 69 h of incubation, the plates were centrifuged, and the medium was replaced with 150 µL of fresh medium containing MTT (0.5 mg. mL^−1^). Following an additional 3 h incubation period, the plates were centrifuged again, and the resulting formazan crystals were dissolved in 150 µL of DMSO. Absorbance was measured at 595 nm using a DTX880 Multimode Detector microplate reader (Beckman Coulter Inc., California, USA).

Cell viability was expressed as a percentage relative to the vehicle-treated control group. The half-maximal inhibitory concentration (IC₅₀) was determined for each cell line by nonlinear regression analysis. Selectivity index (SI) was calculated as the ratio between the IC₅₀ value obtained for the non-tumorigenic L929 cell line and the corresponding IC₅₀ value obtained for each cancer cell line. Based on the cytotoxicity results obtained after 72 h of exposure, the most sensitive cell line was selected for subsequent mechanistic studies conducted at 24 and 48 h.

### Analysis of mechanisms involved in cytotoxic activity

Subsequent experiments were performed to investigate the mechanisms underlying the cytotoxic activity of Oxβ-Lp in NCI-H460 cells. Cells were seeded at a density of 7 × 10^5^ cells/well and exposed to the compound for different experimental periods according to the objectives of each assay. The concentrations selected for the mechanistic studies were based on the IC₅₀ values previously determined and on the extent of cytotoxic damage observed at different exposure times. Doxorubicin (0.05–0.1 μM) was used as a positive control, whereas 0.1% DMSO served as the vehicle control.

### Cell viability assays

The effects of Oxβ-Lp on NCI-H460 cell viability were evaluated using trypan blue exclusion and propidium iodide (PI) staining assays.

For the trypan blue exclusion assay, cells were treated with Oxβ-Lp at concentrations corresponding to ½ × IC₅₀ (3.31 μM), 1 × IC₅₀ (6.62 μM), and 2 × IC₅₀ (13.24 μM) for 24 and 48 h. After treatment, viable and non-viable cells were counted using a Neubauer hemocytometer under a light microscope (Olympus, Tokyo, Japan) [17].

Cell membrane integrity was further evaluated by propidium iodide uptake using flow cytometry. NCI-H460 cells were exposed to Oxβ-Lp at concentrations of ½ × IC₅₀ (3.31 μM), 1 × IC₅₀ (6.62 μM), 3 × IC₅₀ (19.86 μM), and 3.5 × IC₅₀ (23.17 μM) for 24 h. Following treatment, 100 μL aliquots of untreated and treated cell suspensions were incubated with propidium iodide (50 μg/mL) for 5 min at room temperature and analyzed using a Guava EasyCyte Mini® flow cytometer (Guava Technologies, Millipore, Hayward, CA, USA). All experiments were performed in three independent biological replicates, each conducted in quadruplicates.

### Hemolytic activity

Blood samples were collected from Swiss mice (*Mus musculus*) in heparinized tubes. Hemolytic activity was evaluated by assessing the membrane in 96-well plates [18]. Briefly, each well received 100 µL of a 0.85% NaCl solution containing 10 mM CaCl_2_ and 100 µL of a 2% mouse erythrocyte suspension prepared in the same medium. Oxβ-Lp was tested at concentrations ranging from 0.19 to 1.56 μM. 0,5% Triton X-100 was used as a positive control. After incubating for 1 h at room temperature, the plates were centrifuged at 1,500 rpm for 10 min. The absorbance of the released hemoglobin in the supernatant was measured at 540 nm using a Polaris® Microplate reader (São Paulo, SP, Brazil). Hemolytic activity was expressed as a percentage relative to positive control. All animal procedures were approved by the Animal Experimentation Ethics Committee of the Federal University of Piauí (Protocol No. 555/19).

### Cell morphology assays

Morphological alterations induced by Oxβ-Lp in NCI-H460 cells were evaluated using Panoptic staining and acridine orange/ethidium bromide (AO/EB) double staining.

For light microscopy analysis, NCI-H460 cells were cultured on 13 mm circular coverslips and treated with Oxβ-Lp at concentrations corresponding to ½ × IC_50_ (3.31 µM), 1 × IC_50_ (6.62 µM), 3 × IC_50_ (19.86 µM), and 3.5 × IC_50_ (23.17 µM) for 24 h. Following treatment, the cells were fixed with methanol and stained using the Panoptic staining kit (LaborClin, RJ, Brazil). Morphological changes were examined using an Opticam O500R light microscope (Opticam Inc., Technology/Microsoft Windows, Andover, MA, USA), and images were acquired with an UltraK HD Opt5003 Optifocus digital camera.

For fluorescence microscopy analysis, the cells were treated with Oxβ-Lp at concentrations ½ × IC_50_ (3.31 µM), 1 × IC_50_ (6.62 µM), 2 × IC_50_ (13.24 µM), and 2,5 × IC_50_ (16.55 µM) for 24 h. After treatment, the cells were collected by centrifugation, re-suspended in phosphate-buffered saline (PBS), and stained with 1 µL of AO/EB solution (100 µg. mL^−1^).

Viable, apoptotic, and necrotic cells were identified under a fluorescence microscope (Olympus, Tokyo, Japan), and their images were captured using AxioVision Release 4.8 software (Carl Zeiss Microscopy, Thornwood, NY, USA). A total of 300 cells were analyzed per treatment group, and the percentages of apoptotic and necrotic cells were determined as previously described by Cury-Boaventura et al. [19]. Doxorubicin was used as a positive control.

### Mitochondrial membrane potential

Changes in mitochondrial membrane potential were assessed using the fluorescent dye rhodamine 123 as previously described by Cury-Boaventura et al. [19]. NCI-H460 cells were treated with Oxβ-Lp at concentrations of 1 × IC_50_ (6.62 µM), 2 × IC_50_ (13.24 µM), and 2,5 × IC_50_ (16.55 µM) for 24 h. Following treatment, cells were centrifuged at 1,500 rpm for 5 min and re-suspended in 500 µL of rhodamine 123 solution (1 µg/mL). The cell suspensions were incubated at 37 °C for 30 min in the dark. Doxorubicin (Dox) (0.1 µM) was used as a positive control. Fluorescence intensity was measured, and the percentage of mitochondrial depolarization was determined using Guava Express Plus software (Guava Technologies, Millipore, Hayward, CA, USA).

### Cell cycle analysis

Cell cycle distribution was evaluated by flow cytometry following treatment with Oxβ-Lp. NCI-H460 cells were exposed to Oxβ-Lp at concentrations of ½ × IC₅₀ (3.31 μM), 1 × IC₅₀ (6.62 μM), 3 × IC₅₀ (19.86 μM), and 3.5 × IC₅₀ (23.17 μM) for 24 h. After treatment, the cells were incubated for 30 min in the dark with a staining solution containing propidium iodide (50 μg/mL), 0.1% sodium citrate, and 0.1% Triton X-100, according to Nicoletti et al. [20]. Samples were subsequently analyzed using a Guava EasyCyte Mini® flow cytometer (Guava Technologies, Millipore, Hayward, CA, USA). Doxorubicin was used as the positive control. All experiments were performed in three independent biological replicates.

### Comet assay

DNA damage was evaluated using the alkaline comet assay. NCI-H460 cells were treated with Oxβ-Lp at concentrations of 1xIC_50_ (6.62 µM), 2 × IC_50_ (13.24 µM) and 3xIC_50_ (19.86 µM) for 3 h. Following treatment, cells were trypsinized and re-suspended in RPMI medium. An aliquot of 20 µL of the cell suspension was mixed with 100 µL of 0.75% low-melting-point agarose and immediately spread onto a pre-coated slide with 1% normal-melting-point agarose. After agarose solidification, the slides were immersed in a lysis solution containing 2.5 M NaCl, 10 mM Tris–HCl, 100 mM EDTA, 1% Triton X-100, and 10% DMSO (pH 10.0), and maintained at 4 °C overnight.

The slides were then washed with neutralization buffer (0.4 M Tris, pH 7.5) for 5 min and subjected to electrophoresis at 25 V and 300 mA for 20 min. After electrophoresis, the slides were washed with the neutralization buffer, fixed with absolute ethanol, and stained with ethidium bromide (20 µg/mL). Samples were examined under a fluorescence microscope (Olympus, Tokyo, Japan). 100 nuclei per slide were scored according to the extent of DNA migration and classified into five categories, ranging from 0 (no detectable damage) to class 4 (maximum DNA damage). DNA damage index (DI) was calculated for each sample, ranging from 0 (100 cells classified as class 0) to 400 (100 cells classified as class 4), as previously described by Collins [21].

### Genotoxic effects of Oxβ-Lp with *Allium* test

#### *Allium cepa* test

Commercial onion bulbs (*Allium cepa* L.) of uniform size were obtained from a local market in Piauí, Brazil. The bulbs were washed, and old roots, dry outer scales, and central parenchyma were removed to ensure uniform root growth. The assay was performed according to Islam et al. [22], with minor modifications. Groups of five bulbs were placed in chlorine-free water and exposed to Oxβ-Lp at concentrations 0.38, 1.94, 3.88, and 7.78 µM for 48 h at 25 ± 1 °C in the dark. Test solutions were renewed daily. Copper sulfate (0.6 µg. mL⁻^1^) and chlorine-free water served as positive and negative controls, respectively. Root growth (> 3 mm) was measured, and macroscopic alterations were recorded. Root tips were fixed in Carnoy’s solution for 24 h, stored in 70% ethanol, hydrolyzed in 1 N HCl (30 min, 25 °C), rinsed with distilled water, and stained with Schiff’s reagent. Meristematic cells were squashed in 2% acetic carmine and examined under a light microscope at 400 × magnification. Approximately 5,000 cells were examined per treatment group (1,000 cells per slide). Mitotic index (MI) was calculated as follows: MI = (Number of dividing cells/Total number of observed cells) × 100. To assess genotoxic and mutagenic effects, chromosomal aberrations, including chromosomal bridges, chromosome fragments, lagging chromosomes, and chromosome loss, as well as the presence of micronuclei, were recorded during the different stages of mitosis (prophase, metaphase, anaphase, and telophase).

#### Comet assay on the root cells of *Allium cepa*

DNA damage in *Allium cepa* root cells was evaluated using the comet assay as described by Braga et al. [23]. Root tips previously treated and fixed in Carnoy’s solution were mechanically fragmented and incubated overnight in 0.4 M Tris–HCl (pH 7.5) to obtain nuclear suspensions. The nuclei were embedded in 1% low-melting-point agarose and layered onto slides pre-coated with 2.25% normal-melting-point agarose in PBS (pH 7.4). After drying, the slides were subjected to electrophoresis at 25 V, 300 mA, 15 min, fixed in Carnoy’s solution, washed, and stained with 0.02% silver nitrate. Cyclophosphamide (7.66 µM) and chlorine-free water were used as positive and negative controls, respectively. 100 nuclei per treatment were analyzed under a light microscope at 400 × or 1000 × magnification. DNA damage was classified into five categories according to comet tail length: class 0 (no detectable damage), class 1 (low damage), class 2 (moderate damage), class 3 (high damage), and class 4 (maximum damage). DNA damage index (DI) was calculated based on the distribution of nuclei among the different damage classes.

### Acute toxicity test with the *Artemia salina* model

The acute toxicity of Oxβ-Lp was evaluated using the *Artemia salina* lethality bioassay, as previously described [24, 25]. Briefly, *A. salina* eggs were incubated in artificial seawater at 25–30 °C for 48 h to allow hatching. Subsequently, ten viable and active nauplii were transferred to test tubes containing Oxβ-Lp in a final volume of 5 mLOxβ-Lp was dissolved in a DMSO 80 mixture (1:1, v/v; 500 μL) and tested at final concentrations of 7.81, 15.63, 31.25, 62.5, 125.0, and 250.0 μg/mL in artificial seawater. The negative control consisted of DMSO 80 (1:1, v/v; 500 μL) diluted in artificial seawater, whereas the positive control consisted of a 0.1% potassium dichromate (K₂Cr₂O₇) solution. After 24 h of exposure, the numbers of live and dead nauplii were recorded. All experiments were performed in triplicate, and the results were expressed as percentage mortality. The median lethal concentration (LC₅₀) and corresponding 95% confidence intervals were estimated by Probit analysis.

Toxicity classification was based on the criteria proposed by McLaughlin et al. [25], according to which compounds are classified as non-toxic (LC₅₀ > 1000 μg/mL), low toxicity (LC₅₀ = 500–1000 μg/mL), moderate toxicity (LC₅₀ = 100–500 μg/mL), or highly toxic (LC₅₀ < 100 μg/mL).

### Statistical analysis

Data are presented as mean ± standard error of the mean (SEM). Statistical analysis and graphical representations were performed using the OriginPRO® 2025 Program (OriginLab® Corporation, MA, USA).

Comparisons among multiple groups were performed using one-way analysis of variance (ANOVA), followed by the appropriate post hoc test (Tukey’s, Dunnett’s, or Bonferroni’s multiple-comparison test) according to the experimental design. Differences were considered statistically significant when p < 0.05. All statistical tests were performed using a 95% confidence level.

### Physicochemical and ADMET properties

The physicochemical properties of Oxβ-Lp and its precursor β-lapachone (β-Lp) were predicted using the online software SwissADME web server (http://www.swissadme.ch/) [26]. Parameters related to absorption, distribution, metabolism, excretion, and toxicity (ADMET) were estimated using the online platforms PreADMET (https://preadmet.qsarhub.com/) and pkCSM (http://biosig.unimelb.edu.au/pkcsm/) [27].

### Molecular docking

Molecular docking analyses were performed to investigate the potential interaction of Oxβ-Lp and its precursor β-lapachone (β-Lp) with NAD(P)H oxidoreductase 1 (NQO1), a key enzyme involved in quinone bioactivation and redox cycling [28]. The three-dimensional structures of Oxβ-Lp and β-Lp were generated using ChemSketch software and exported in MOL format. The crystal structure of human NQO1 (PDB ID: 2F1O) was retrieved from the Protein Data Bank (https://www.rcsb.org/) [29]. Protein and ligand structures were prepared and optimized using the ArgusLab 4.0.1 program. Water molecules, ions, co-crystallized ligands, and protein chains not directly involved in ligand binding were removed from the receptor structure. The AC and BD dimers were selected as docking sites, while flavin adenine dinucleotide (FAD) molecules located within the catalytic pockets were retained to preserve the structural integrity of the active sites. Docking calculations performed using the ArgusDock exhaustive search algorithm implemented in ArgusLab and evaluated using the AScore scoring function. Docking simulations were carried out with a grid resolution of 0.40 Å, employing “Regular Accuracy” settings and flexible ligand conformational sampling.

For each compound, binding poses exhibiting the lowest AScore values (highest predicted binding affinity) within the AC and BD catalytic dimers were selected for further analysis. Protein–ligand interactions were characterized based on interaction energy, binding orientation, and the types of intermolecular interactions involved.

Three-dimensional visualization and two-dimensional interaction maps of the protein–ligand complexes were generated using BIOVIA Discovery Studio Visualizer. To validate the docking protocol and assess its reliability and reproducibility, the native ligand dicoumarol (DIC) was re-docked into the NQO1 binding site, and the resulting binding pose was compared with the crystallographic conformation.

## Results and discussion

### In vitro cytotoxicity

Oxβ-Lp exhibited cytotoxic activity against all tested cancer cell lines after 72 h of exposure, with IC₅₀ values ranging from 1.88 to 15.19 μM (Table 1). Among the evaluated cell lines, NCI-H460 non-small cell lung cancer cells were the most sensitive to treatment, displaying the lowest IC₅₀ value (1.88 μM) and the highest selectivity index (SI = 13.1). Although Oxβ-Lp was less potent than doxorubicin, it exhibited a more favorable selectivity profile in NCI-H460, K562, and PC9 cells, suggesting reduced toxicity toward non-tumor cells.

**Table 1:** Cytotoxicity and selectivity index of Oxβ-Lp in human cancer cell lines and non-tumoral cell lines

Cell lines	IC_50_ (µM)		SI	
	Oxβ-Lp	Dox	Oxβ-Lp	Dox
NCI-H460	1.88 (1.66–2.13)	0.28 (0.24–0.33)	13.10	6.14
K562	4.73 (4.04–5.53)	0.46 (0.45–0.47)	5.20	4.09
PC9	7.43 (5.46–10.12)	0.59 (0.48–0.70)	3.31	2.91
HEPG2	15.19 (13.34–17.3)	0.61 (0.53–0.68)	1.62	2.81
L929	24.63^a^ (21.13–28.73)	1.72 (1.58–1.87)	ND	ND

*ND*, Not determined

IC_50_ values are presented as mean ± standard deviation and 95% confidence interval. Doxorubicin (Dox) was used as a positive control

^a^Araújo et al. [15]

The cytotoxic activity observed in the present study is consistent with previous findings reported for this derivative in SF-295 (central nervous system), HCT-116 (colon carcinoma), NCI-H1975 (lung carcinoma), and HL-60 (leukemia) cell lines, supporting the broad antitumor potential of Oxβ-Lp [15].

Interestingly, the highest sensitivity was observed in NCI-H460 cells. Previous studies have reported elevated an endogenous NQO1 expression in this cell line; however, NQO1 expression and activity were not evaluated in the present study. Therefore, the factors underlying the enhanced susceptibility of NCI-H460 cells to Oxβ-Lp remain to be fully elucidated. In studies involving naphthoquinone derivatives, the most selective compound for the NCI-H460 cell line exhibited an SI of 10.69 against human embryonic fibroblast (MRC-5) and an IC_50_ of 0.159 µM [30]. The SI is a critical parameter for estimating drug safety and therapeutic potential, with values greater than 3 generally indicating selective cytotoxicity [31].

The precursor molecule of Oxβ-Lp, β-Lapachone (β-Lp), has previously demonstrated potent cytotoxic activity against HEPG2 cells, with IC_50_ of 2.38 μM at 72 h of exposure [32], while exhibiting low cytotoxicity in L929 cells after 98 h of treatment [33]. Moreover, β-Lp displayed high selectivity toward HEPG2 and K562 cells compared to non-tumoral human lung fibroblast MRC-9 cells (SI 37 and 29, respectively) [34].

Although direct comparative experiments between β-lapachone and Oxβ-Lp were not performed under identical experimental conditions, relevant differences can be inferred from previously published studies. Compared with the cytotoxicity profiles reported for β-lapachone, Oxβ-Lp exhibited marked selectivity toward NCI-H460, together with lower hemolytic activity at cytotoxic concentrations. These observations suggest that oxime derivatization may influence not only cytotoxic potency but also selectivity and toxicity characteristics of the parent naphthoquinone scaffold.

β-Lapachone is known to induce apoptosis through mechanisms associated with NAD(P)H oxidoreductase 1 (NQO1)-mediated bio-activation and has shown considerable therapeutic potential against several tumor types exhibiting elevated endogenous NQO1 expression, including non-small cell lung cancer (NSCLC) [9, 35]. Consistent with this mechanism, Torrente et al. [35], demonstrated that NCI-H460 cells harboring NQO1 mutations exhibited resistance to β-lapachone treatment.

Interestingly, NCI-H460 cells were the most sensitive cell line evaluated in the present study. Previous reports have described elevated endogenous NQO1 expression in this cell line, which may contribute to the enhanced susceptibility of NCI-H460 cells to β-lapachone derivatives. However, NQO1 expression and enzymatic activity were not directly assessed in the present study. Therefore, any association between Oxβ-Lp sensitivity and NQO1 status should be interpreted with caution. The redox cycling mediated by NQO1 promotes excessive reactive oxygen species (ROS) generation, DNA damage, and PARP-1 hyper-activation, ultimately leading to NAD⁺/ATP depletion and programmed cell death, as previously described in NSCLC models [10]. In addition, β-lapachone has been reported to exert anti-tumoral effects through modulation of survival-related signaling pathways, including Akt/mTOR inhibition, particularly in tumor cells presenting elevated endogenous NQO1 expression [10, 11, 36–38]. Considering the close structural similarity between β-lapachone and Oxβ-Lp, some biological effects reported for β-lapachone may provide useful hypotheses for future mechanistic investigations. However, the molecular pathways responsible for the cytotoxic activity of Oxβ-Lp were not directly evaluated in the present study.

The pronounced sensitivity of NCI-H460 cells to Oxβ-Lp may be related to biological characteristics intrinsic to this cell line [38–40]. Although elevated endogenous NQO1 expression has previously been reported in NCI-H460 cells, the contribution of this enzyme to Oxβ-Lp sensitivity was not investigated in the present study and therefore remains unknown. Notably, the high selectivity index observed for Oxβ-Lp in NCI-H460 cells highlights the potential of this naphthoquinone derivative as a promising candidate for the treatment of non-small cell lung cancer (NSCLC).

This finding is particularly relevant considering that lung cancer remains one of the most commonly diagnosed malignancies and the leading cause of cancer-related mortality worldwide [41]. NSCLC accounts for approximately 80–85% of all new lung cancer cases [42] and is frequently associated with poor clinical outcomes, high recurrence rates, and limited therapeutic response in advanced stages [43, 44]. Therefore, the identification of novel compounds exhibiting selective cytotoxicity toward NSCLC cells remains an important objective in anticancer drug development.

### Evaluation of the cytotoxicity mechanism

**Fig. 2:**
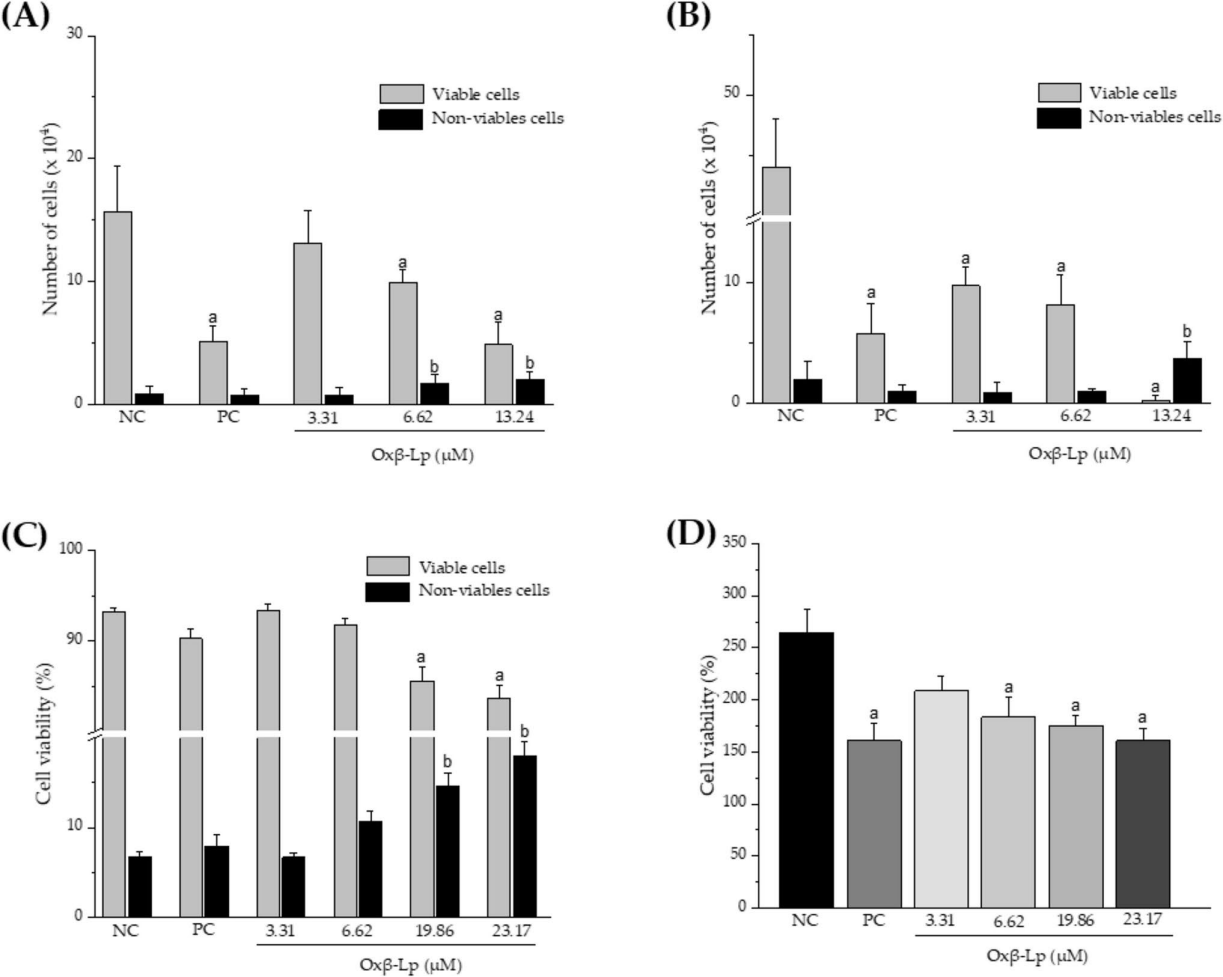
Anti-proliferative effect of Oxβ-Lp in lung cancer cells (NCI-H460) by trypan blue test at 24 h (**A**) and 48 h (**B**); % cell viability assessed by membrane integrity test at 24 h (**C**), cell density obtained in membrane integrity test at 24 h (**D**). a. *p* < 0.05 compared with negative control (NC); b. *p* < 0.05 compared with positive control (PC, Doxorubicin at 0.1 µM)

The MTT assay results supported the selection of the NCI-H460 cell line for subsequent mechanistic studies. Based on these experiments, the IC_50_ values for Oxβ-Lp were determined to be 6.4 µM and 6.8 µM after 24 h and 48 h of exposure, respectively, and these concentrations were used to guide the design of subsequent assays. Consistent with the MTT findings, the trypan blue exclusion assay confirmed the anti-proliferative effect of Oxβ-Lp. Treatment with 6.62 µM significantly reduced the number of viable NCI-H460 cells after 24 h of exposure (Fig. 2A). Moreover, all tested concentrations decreased cell viability, with a more pronounced effect observed after 48 h of treatment (Fig. 2B). These results indicate that Oxβ-Lp exerts time- and concentration-dependent inhibitory effects on the viability and proliferation of NCI-H460 cells. Similar findings have been reported for other naphthoquinone derivatives, which induced concentration-dependent reductions in cell viability after 24 h of exposure [45, 46].

Flow cytometry analysis demonstrated a significant reduction in NCI-H460 cell viability only at the highest concentrations of Oxβ-Lp (19.8 and 23.1 µM) after 24 h of exposure (Fig. 2C). This finding suggests that the early anti-proliferative effects of Oxβ-Lp are not primarily associated with loss of plasma membrane integrity. Consistent with this observation, a progressive decrease in cell density was also observed with increasing concentrations of Oxβ-Lp (Fig. 2D), corroborating the anti-proliferative effects previously detected by the trypan blue assay.

The selectivity of Oxβ-Lp was further supported by the hemolysis assay. Oxβ-Lp induced hemolytic damage in mouse erythrocytes only at substantially high concentrations [EC_50_ 483.4 µM (377.9–618.0 µM)], than those required to exert cytotoxic effects in cancer cell lines. These findings indicate that the cytotoxic activity of Oxβ-Lp is unlikely to result from nonspecific membrane disruption. Previous studies have reported that β-lapachone induces hemolysis at concentrations ranging from 10 to 30 µM, which overlap with its antitumor concentrations [47]. Additionally, hemolytic anemia was described as one of the dose-limiting toxicities associated with β-lapachone in clinical studies [48]. Although direct comparative experiments between β-lapachone and Oxβ-Lp were not performed under identical experimental conditions, the markedly lower hemolytic activity observed for Oxβ-Lp suggests a potentially improved selectivity profile.

Structural modification through oximation may alter physicochemical and membrane interaction, cellular uptake, thereby influencing toxicity and selectivity profiles [49, 50]. In this context, the reduced hemolytic activity observed for Oxβ-Lp compared with literature reports for β-lapachone may indicate a partial reduction in nonspecific membrane-associated toxicity. Nevertheless, direct comparative studies will be necessary to confirm whether oxime derivatization effectively improves the therapeutic window of β-lapachone derivatives.

**Fig. 3:**
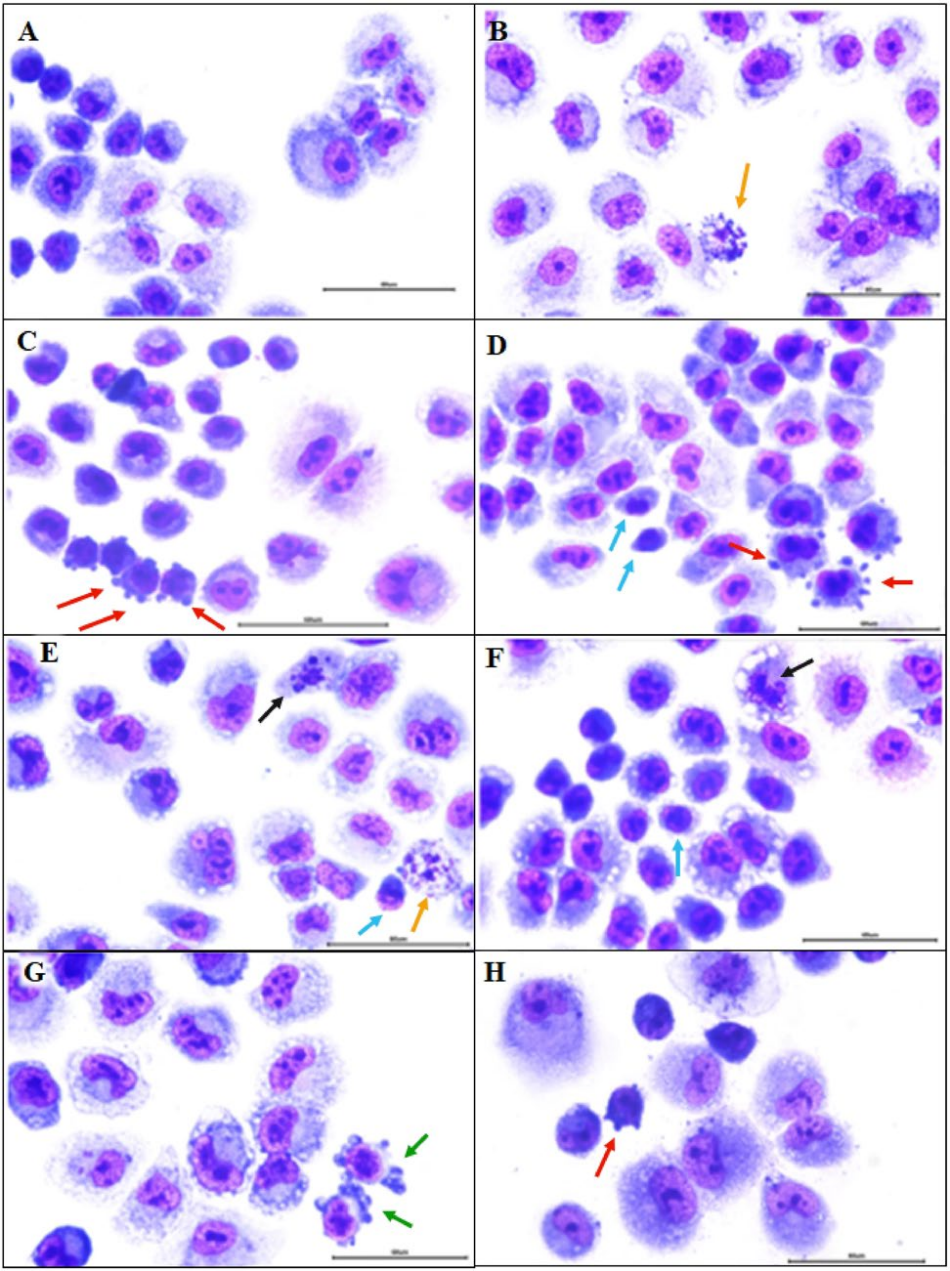
Morphology of NCI-H460 cells treated with Oxβ-Lp for 24 h, observed using panoptic staining. Negative control (**A**); Positive control, Doxorubicin 0.5 µM (**B**); Oxβ-Lp [3.31 µM (**C**); 6.62 µM (**D**); 13.24 µM (**E**); 16.55 µM (**F**); 19.86 µM (**G**); 23.17 µM (**H**)]. Apoptotic bodies (red arrow); nuclear fragmentation (black arrow); blebs (green arrow); chromosome condensation (blue arrow); cell remnants (orange arrow). Scale bar: 60 µm

Microscopic examination of treated and untreated NCI-H460 cells revealed marked morphological alterations following Oxβ-Lp exposure at all tested concentrations after 24 h (Fig. 3). In addition, the images illustrate the characteristic pleomorphic morphology of this cell line. These observations provide further evidence of the cytotoxic effects induced by Oxβ-Lp and support the quantitative findings obtained in the viability assays.

In doxorubicin-treated cells, extensive cellular debris was observed, consistent with severe cytotoxic damage (Fig. 3B). Exposure to Oxβ-Lp induced morphological alterations characteristic of apoptosis, including chromatin condensation, nuclear fragmentation, membrane blebbing, and the formation of apoptotic bodies (Fig. 3 C–H). At the highest concentration tested (23.17 μM), some cells exhibited nuclear swelling and increased volume (Fig. 3H), morphological features commonly associated with necrotic cell death. These observations are consistent with the increased loss of membrane integrity detected by propidium iodide staining at higher concentrations.

**Fig. 4:**
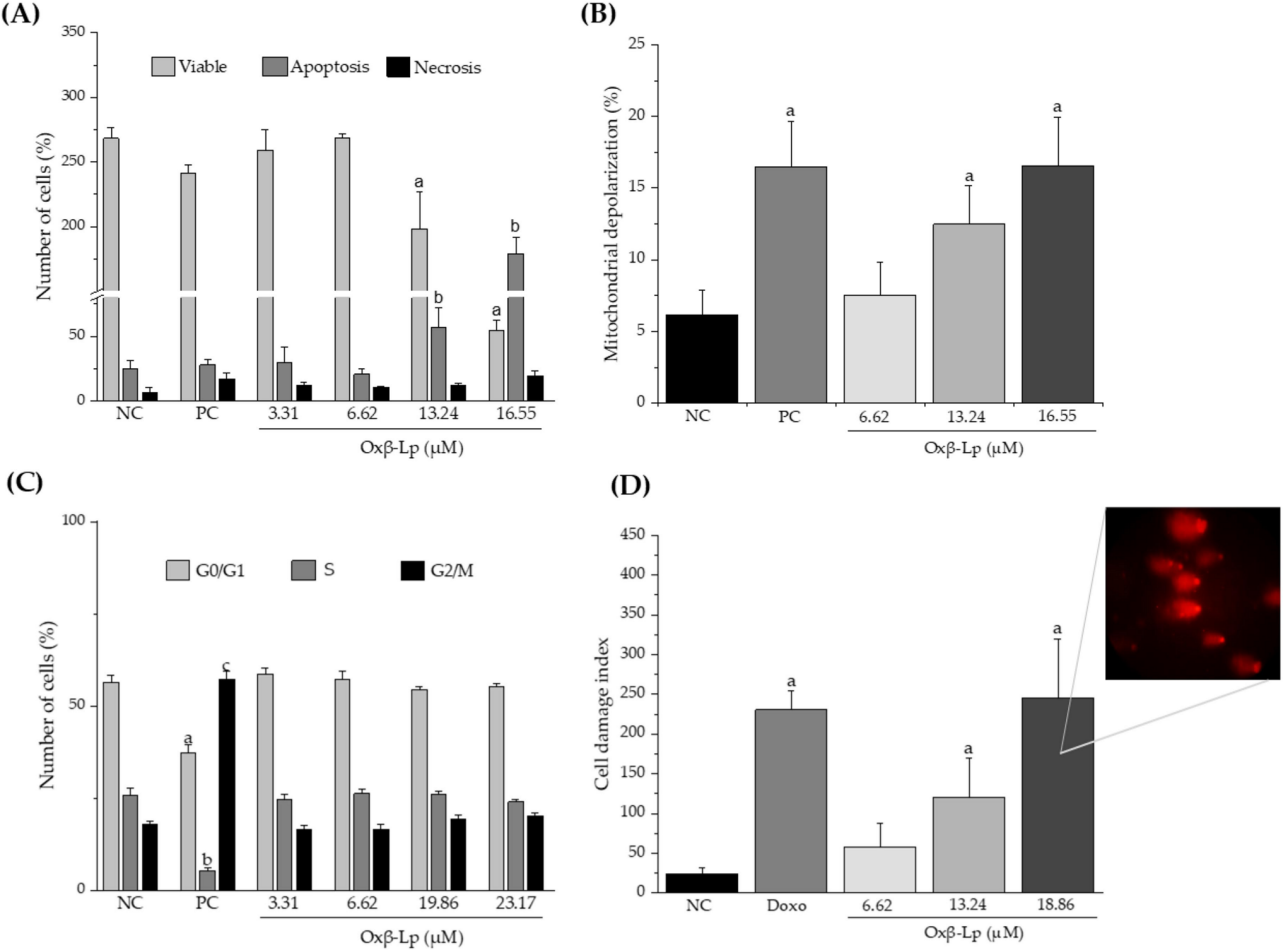
Effects of cytotoxicity and genotoxicity induced by Oxβ-Lp in the NCI-H460 cell line. **A** Morphological changes were evaluated by AO/EB differential fluorescent labeling after 24 h of treatment. a. *p* < 0.05 compared with NC; b. *p* < 0.05 compared with PC. **B** Mitochondrial depolarization was observed through rhodamine 123 after 24 h of treatment. a. *p* < 0.05 compared with NC; **C** Effect on the cell cycle phases after 24 h of treatment. NC (negative control); PC (positive control, Doxorubicin at 0.1 µM). a (G0/G1), b (S), c (G2/M): significantly different from the NC *p* < 0.05); **D** Cell damage index after 3 h of treatment by the comet assay and comet tails in class 3 observed at 19.8 μM concentration (magnification × 400). a. *p* < 0.05 compared with NC

Apoptotic cells typically maintain plasma membrane integrity throughout most stages of the death process, whereas necrotic cells are characterized by cellular swelling and membrane rupture [51]. The apoptotic morphology observed by light microscopy was further supported by acridine orange/ethidium bromide (AO/EB) staining (Fig. 4A), which confirmed the predominance of apoptotic features following Oxβ-Lp treatment. Collectively, these findings suggest that apoptosis represents the major mode of cell death induced by Oxβ-Lp at cytotoxic concentrations, whereas necrotic events become more evident at higher exposure levels.

Similar apoptotic morphological patterns have been reported for β-lapachone and other naphthoquinone derivatives in lung cancer cells, reinforcing the involvement of programmed cell death pathways in the antitumor activity of this class of compounds [52].

AO/EB differential staining revealed a significant increase in the proportion of apoptotic cells and a concomitant reduction in viable cells following treatment with Oxβ-Lp at 13.24 μM. In contrast, no significant increase in necrotic cells was observed at any of the concentrations tested. These findings indicate that apoptosis is the predominant mode of cell death induced by Oxβ-Lp under the experimental conditions employed. Similar results have been reported for β-lapachone, which induces apoptosis in NSCLC cell lines, including H1299 and NCI-H358 [53].

Mitochondrial membrane depolarization is recognized as an early hallmark of apoptosis and reflects disruption of mitochondrial function during the activation of intrinsic cell death pathways [54, 55]. Therefore, mitochondrial transmembrane potential was evaluated by measuring rhodamine 123 incorporation using flow cytometry (Fig. 4B). Oxβ-Lp induced mitochondrial depolarization of 12.48% and 15.05% at concentrations of 13.24 µM and 16.55 µM, respectively.

The observed loss of mitochondrial membrane potential supports the hypothesis that Oxβ-Lp induces apoptosis, at least in part, through activation of the intrinsic mitochondrial pathway. Cellular stressors, such as oxidative damage and DNA lesions, can trigger mitochondrial outer membrane permeabilization, leading to dissipation of the electrochemical gradient and activation of downstream apoptotic signaling cascades [55].

Several studies have demonstrated that β-lapachone and related naphthoquinones promote apoptosis through mechanisms involving reactive oxygen species (ROS) generation, mitochondrial dysfunction, and oxidative DNA damage [56–58]. In addition to oxidative stress-mediated effects, β-lapachone has also been associated with the modulation of signaling pathways involved in cell survival, proliferation, and metastasis, including Akt/mTOR modulation [59–62].

The cytotoxic activity of β-lapachone is classically associated with NAD(P)H oxidoreductase 1 (NQO1)-mediated bio-activation, which promotes redox cycling and excessive ROS generation [7]. Previous studies have reported elevated endogenous NQO1 expression in tumor types, where this enzyme has been associated with tumor progression and poor clinical outcomes [63]. Although NQO1 expression and activity were not evaluated in the present study, the pronounced sensitivity of NCI-H460 cells to Oxβ-Lp, together with the observed mitochondrial dysfunction, DNA damage, and apoptotic phenotype, indicates that multiple intracellular pathways may contribute to its cytotoxic effects. Further studies will be necessary to define the molecular targets and signaling pathways involved. Therefore, the mechanism of action of Oxβ-Lp is likely multifactorial and may not be exclusively dependent on ROS generation.

Further studies investigating intracellular ROS production, NQO1 activity, and signaling pathways such as Akt/mTOR will be necessary to better define the molecular mechanisms underlying the antitumor activity of Oxβ-Lp.

Figure 4C illustrates the effects of the Oxβ-Lp compound on the cell cycle phases in NCI-H460 cells. DNA damage is generally associated with cell cycle arrest checkpoints, leading to transient or sustained cell cycle arrest to allow DNA repair [64]. However, this pattern was not observed following Oxβ-Lp treatment. As shown in Fig. 4C, no significant accumulation of cells was detected in any specific phase of the cell cycle after exposure to Oxβ-Lp. In contrast, doxorubicin treatment resulted in a marked increase in the proportion of cells in the G_2_/M phase, accompanied by a reduction in the S phase population, consistent with its well-established mechanism of action.

Cellular responses to DNA damage are highly dependent on cell type, genetic background, and the extent of genomic injury. While moderate DNA damage may trigger cell cycle arrest and DNA repair mechanisms, extensive or irreparable damage often redirects cells toward apoptosis or senescence [65]. In the present study, despite evidence of DNA damage and mitochondrial dysfunction, Oxβ-Lp did not significantly alter cell cycle progression, suggesting that its cytotoxic effects are primarily associated with the activation of cell death pathways rather than checkpoint-mediated growth arrest.

These findings are consistent with previous studies reporting that β-lapachone induces cytotoxicity independently of cell cycle phase in MCF-7 and T47D cell lines, without causing accumulation at specific cell cycle checkpoints [9]. Accordingly, the anti-proliferative activity of Oxβ-Lp appears to result predominantly from apoptosis induction rather than direct modulation of cell cycle regulatory machinery.

Previous investigations have shown that β-lapachone and other naphthoquinone derivatives can induce cell cycle delay or arrest in the S and G_2_/M phases in certain cancer cell types [34, 66, 67]. However, the effects of quinones on cell cycle progression appear to be highly context-dependent. Similar to the results observed for Oxβ-Lp, 2-methoxy-1,4-naphthoquinone has been reported to induce apoptosis in A549 lung cancer cells without promoting significant cell cycle arrest. In that model, cell death was associated with activation of the JNK and p38 MAPK pathways in response to oxidative DNA damage generated by reactive oxygen species (ROS) [68].

DNA damage is a common feature of quinone-induced cytotoxicity and is frequently associated with oxidative stress resulting from redox cycling [5, 69]. Nevertheless, naphthoquinones may also trigger alternative genotoxic mechanisms, including histone H2AX phosphorylation, topoisomerase inhibition, and disruption of DNA replication processes, all of which ultimately converge on apoptotic cell death pathways [70–72]. Collectively, these findings support the hypothesis that DNA damage contributes to the cytotoxic activity of Oxβ-Lp, although the precise molecular events linking genotoxic stress and apoptosis remain to be elucidated.

To further investigate the genotoxic potential of Oxβ-Lp, NCI-H460 cells were exposed to the compound for 3 h and subsequently analyzed using the comet assay. As shown in Fig. 4D, Oxβ-Lp induced significant DNA damage at the highest concentrations tested, whereas the positive control (doxorubicin) produced extensive DNA fragmentation. Representative comet images further illustrate the degree of nuclear DNA damage in treated cells (Fig. 4D).

The induction of DNA damage is consistent with the proposed mechanisms of action of quinone-based antitumor agents. Doxorubicin, for example, intercalates into DNA, inhibits topoisomerase II activity, and disrupts DNA repair processes, ultimately leading to genomic instability and cell death [73, 74]. Similarly, vosaroxin, a first-in-class quinolone antineoplastic agent evaluated for the treatment of acute myeloid leukemia, acts through DNA intercalating DNA intercalation and topoisomerase II inhibition, inducing double-strand DNA breaks, G_2_/M arrest, and apoptosis [75]. Together, these findings support the concept that quinone- and quinolone-derived compounds may exert antitumor activity not only through oxidative stress-mediated mechanisms but also through direct disruption of DNA integrity.

### Cytotoxic and genotoxic evaluation in *Allium cepa* cells

**Fig. 5:**
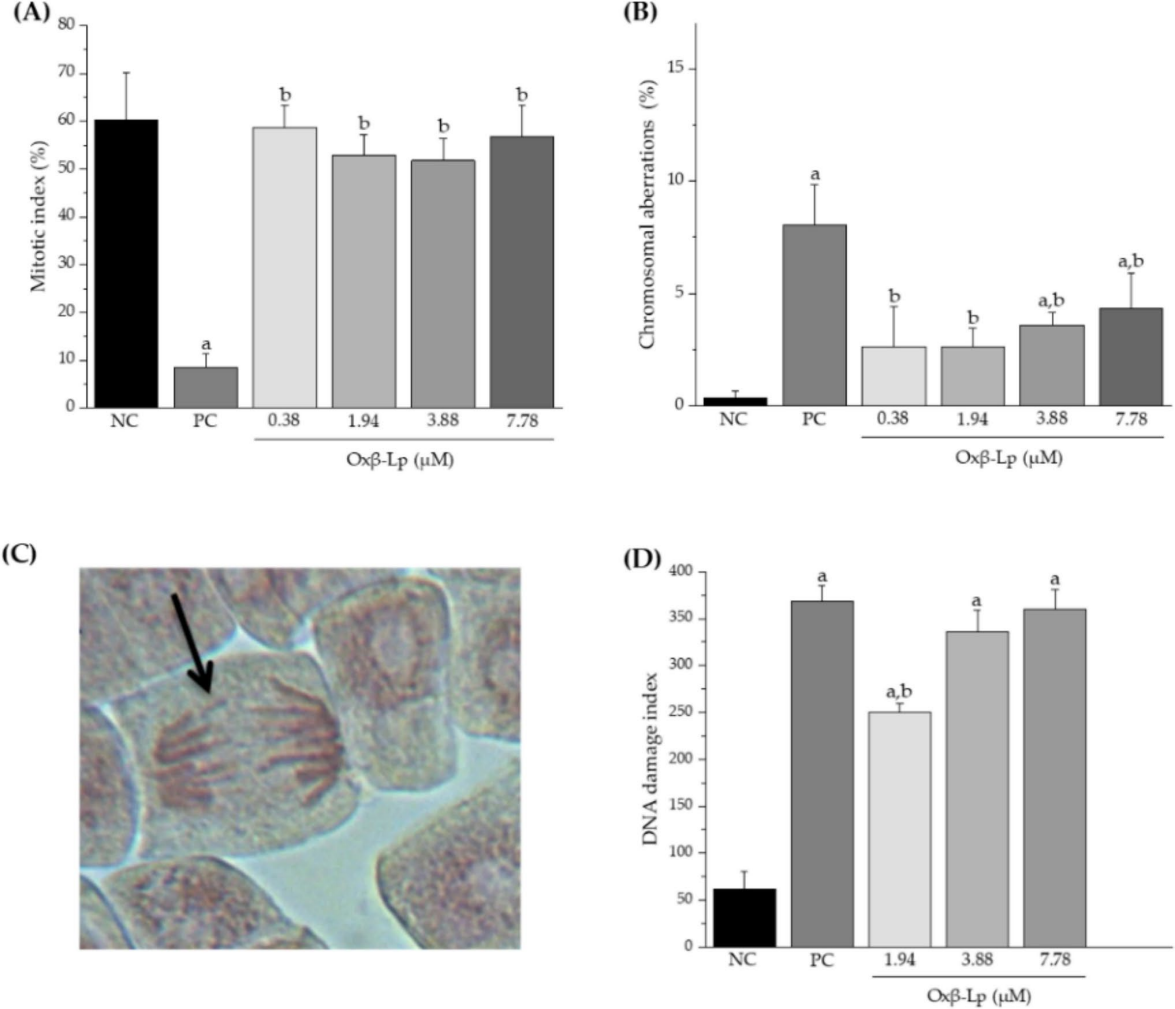
Cytotoxic and genotoxic activity of Oxβ-Lp in the meristematic cells of *Allium cepa* roots after 48 h. **A** Mitotic index (%) of the average of 1,000 cells treated at different concentrations; **B** Chromosomal cell aberrations observed at varying concentrations; **C** Chromosomal fragments (black arrow) after 48 h of exposure to Oxβ-Lp at a concentration of 7.78 μM (magnification × 1000); **D** Index of DNA damage in the cells by the comet test. In A and B: a. *p* < 0.05 compared with negative control (NC); b. *p* < 0.05 compared with positive control (PC, Copper sulfate). In D: a. *p* < 0.05 compared with negative control (NC); b. *p* < 0.05 compared with positive control (PC, Cyclophosphamide at 7.66 µM)

The cytotoxic and genotoxic effects of Oxβ-Lp were further evaluated in meristematic root cells of *Allium cepa* following 48 h of exposure (Fig. 5). Quantification of cells in interphase and mitosis revealed no statistically significant differences compared with the negative control, indicating the absence of detectable cytotoxicity at the concentrations tested (Fig. 5A).

In contrast, a significant increase in chromosomal aberrations was detected at 3.88 and 7.78 µM (Fig. 5B) although the frequency of abnormalities remained lower than that observed in the positive control group. These findings indicate that Oxβ-Lp exhibits genotoxic effects under conditions that do not substantially impair cell proliferation.

Similar results have been reported for β-Lp, which reduced the mitotic index of *A. cepa* meristematic cells only at relatively high concentrations (100 µM) after 18 h of exposure [76]. Therefore, the absence of significant cytotoxicity observed in the present study may be related to the lower concentrations employed, whereas the detected chromosomal alterations suggest that genotoxic effects can occur independently of overt cytotoxicity.

Figure 5D illustrates representative chromosomal fragments observed in *Allium cepa* meristematic cells exposed to Oxβ-Lp at 7.78 μM. The chromosomal abnormalities identified during microscopic analysis included chromosomal bridges, lagging chromosomes, chromosome fragments, and chromosome loss illustrated in Fig. S1.

Genotoxicity compounds evaluated using the *A. cepa* assay frequently induce both chromosomal damage and cytotoxic effects [77]. However, although Oxβ-Lp significantly increased the frequency of chromosomal aberrations, these alterations were not accompanied by a reduction in the mitotic index. This finding suggests that the compound primarily affects genomic integrity without substantially impairing cell proliferation under the experimental conditions tested. Moreover, the absence of micronucleus formation in all treatment groups indicates that Oxβ-Lp did not exhibit detectable mutagenic activity in this model.

**Fig. 6:**
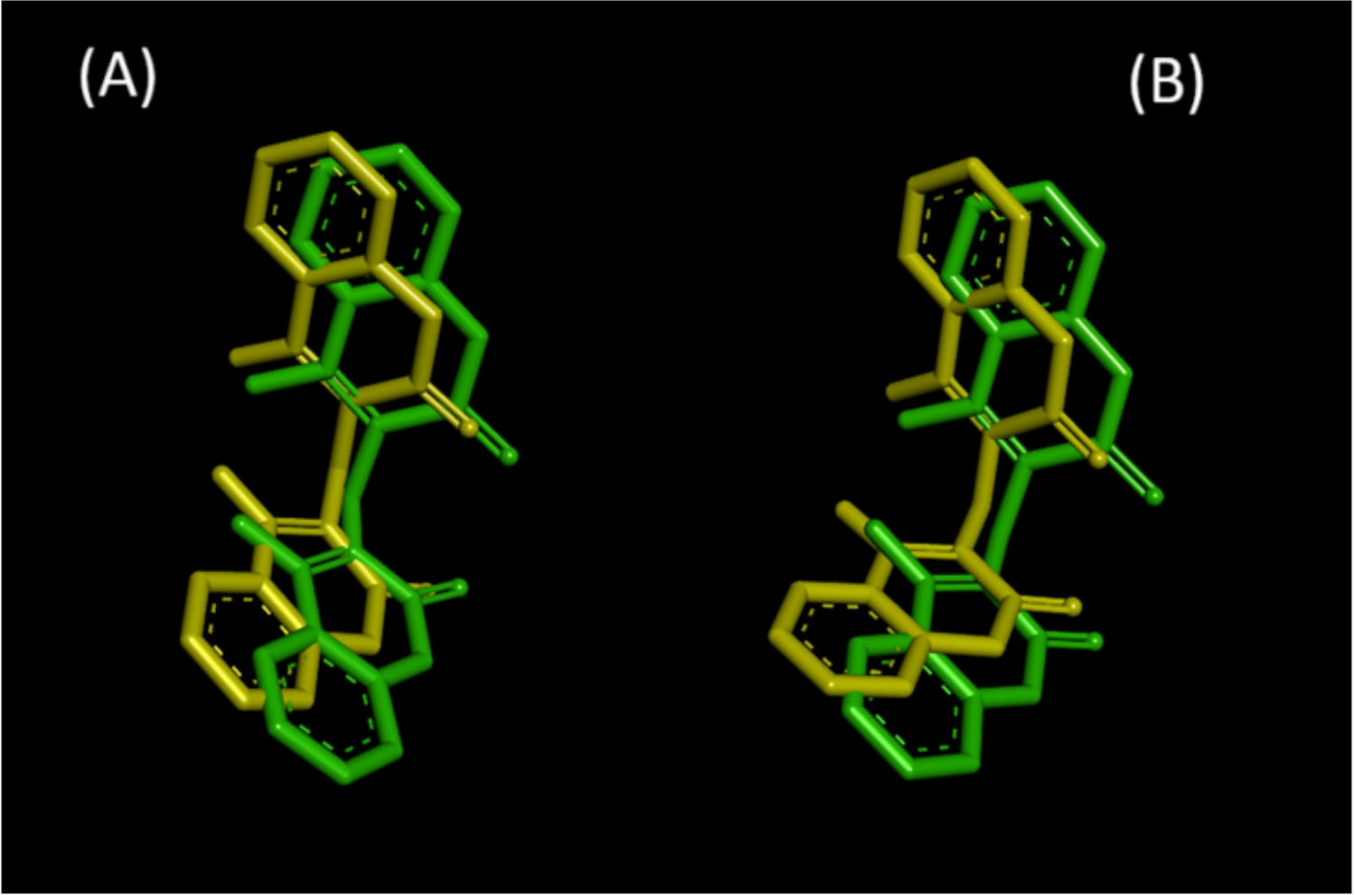
Superimposing of the docked pose over the reference pose of the DTC ligand: **A** Docking using AC dimer; **B** Docking using BD dimer. The original pose is indicated by green, and the re-docked pose is indicated by yellow

To further characterize the genotoxic effects of Oxβ-Lp, DNA damage was evaluated using the comet assay, a highly sensitive method for detecting DNA breaks in *A. cepa* meristematic cells [21]. This assay has been widely employed in environmental and pharmacological genotoxicity studies [23, 78, 79]. As shown in Fig. 6C, treatment with Oxβ-Lp at concentrations of 1.91, 3.88, and 7.78 μM significantly increased the DNA damage index (DI), corroborating the chromosomal aberration findings.

Notably, the DNA damage induced by Oxβ-Lp was comparable to that observed with cyclophosphamide, a well-established DNA-alkylating antineoplastic agent that exerts its cytotoxicity effects through the induction of DNA lesions [80]. Similarly, genotoxic responses have also been reported for clinically relevant chemotherapeutic agents, including 5-fluorouracil, capecitabine, cisplatin, and doxorubicin, in the *A. cepa* model [81].

Taken together, these findings demonstrate that Oxβ-Lp can induce significant DNA damage in both mammalian tumor cells and plant meristematic cells. Although the precise relationship between genotoxicity and cytotoxicity remains to be established, the observed DNA damage may contribute, at least in part, to the antitumor effects of this compound.

Because genotoxicity may be accompanied by systemic toxicity, the toxicological profile of Oxβ-Lp was subsequently investigated using the *Artemia salina* lethality assay.

### Acute toxicity testing on *Artemia salina*

Oxβ-Lp exhibited an LC_50_ value of 16.80 µg. mL^−1^ (95% CI: 14.54–19.79), classifying it as highly toxic according to the criteria proposed by McLaughlin’s scale (LC₅₀ < 100 µg. mL⁻^1^) [82]. Lethality assay is widely used as an initial screening tool for evaluating the toxicological potential of bioactive compounds because of its simplicity, low cost, and good correlation with both in vitro cytotoxicity assays and in vivo toxicity models [83].

Previous studies have reported pronounced toxicity for several naphthoquinones and related derivatives, including 2-hydroxy-[1,4]naphthoquinone and structurally related compounds [84]. Therefore, the toxicity observed for Oxβ-Lp is consistent with the known biological profile of this chemical class. The potent cytotoxic activity displayed by many naphthoquinones is frequently accompanied by toxicological liabilities associated with oxidative stress, redox cycling, and nonspecific cellular damage [85, 86].

Although the high toxicity observed in the *A. salina* model indicates the need for further toxicological evaluation, it should be interpreted in the context of the marked selectivity demonstrated by Oxβ-Lp toward NCI-H460 cells and its comparatively low hemolytic activity at cytotoxic concentrations. Together, these findings suggest that structural modification through oximation may preserve antitumor activity while partially improving the selectivity profile of the parent molecule.

### Physicochemical properties and ADMET prediction

In silico predictions of absorption, distribution, metabolism, excretion, and toxicity (ADMET) parameters have become an important strategy in the early stages of drug discovery, allowing the identification of physicochemical and pharmacokinetic characteristics associated with favorable drug development profiles [87].

The SwissADME predictions for Oxβ-Lp and β-lapachone, including physicochemical parameters, lipophilicity, pharmacokinetics, drug-likeness, medicinal chemistry descriptors, and BOILED-Egg analysis, are presented in Figs. S2 and S3.

Both molecules have molecular weights ≤ 500 g/mol, meeting a requirement for promising oral drug candidates[88]. Oxβ-Lp exhibited higher water solubility (logS), polarity (TPSA), molar refractivity (MR), and a larger number of hydrogen bond donors and acceptors. These changes suggest that oxime derivatization may improve physicochemical properties relevant to drug absorption and distribution.

Furthermore, both compounds exhibited TPSA values below 140 Å and LogP < 5, indicating physicochemical characteristics generally associated with favorable oral bioavailability [88, 89]. Consistent with these findings, the SwissADME bioavailability radar indicated that both molecules fall within the optimal physicochemical space for orally administered drugs.

The bioavailability radar obtained in SwissADME was used to enhance the understanding of how predicted physicochemical parameters are desirable attributes for an effective oral drug [90]. Despite slight differences in the oral bioavailability parameters of both molecules, they fall within the pink area of the graph, indicating an appropriate physicochemical environment for orally administered drugs [89]. The pharmacokinetic parameters for both drugs were similar. Drug-likeness analysis demonstrated that Oxβ-Lp retained a pharmacological profile comparable to that of β-lapachone, satisfying the Lipinski, Ghose, Veber, Egan, and Muegge criteria without any rule violations. These results suggest that oxime derivatization did not compromise the drug-like properties of the parent scaffold.

The BOILED-Egg model (Fig. S4) predicted efficient gastrointestinal absorption for both compounds, represented by their localization within the white region of the diagram. In addition, both molecules were predicted to penetrate the blood–brain barrier, as indicated by their position within the yellow region. Neither compound was predicted to be a substrate of P-glycoprotein (P-gp), suggesting a reduced likelihood of active efflux-mediated transport [89, 91]. Collectively, these predictions indicate that Oxβ-Lp combines favorable drug-likeness characteristics with physicochemical properties that may support oral administration and tissue distribution, warranting further pharmacokinetic and in vivo investigations.

As shown in Table S1, both Oxβ-Lp and β-lapachone were predicted to exhibit high intestinal absorption (> 95%) and high permeability in MDCK cells (> 70 nm/s), suggesting favorable characteristics for oral absorption [92]. In addition, neither compound was predicted to act as a substrate or inhibitor of P-glycoprotein (P-gp/MDR1), indicating a reduced likelihood of active efflux-mediated transport and multidrug resistance-related limitations.

A notable difference between the compounds was observed in plasma protein binding. Oxβ-Lp exhibited a lower predicted plasma protein binding rate (85.90%) than β-Lp (98.28%). Reduced plasma protein binding may increase the fraction of free drug available for tissue distribution and pharmacological activity, potentially influencing its biological effects. Furthermore, both compounds were predicted to cross the blood–brain barrier (LogBB >  − 1) although Oxβ-Lp exhibited a more favorable predicted BBB permeability profile according to the criteria proposed by Pires et al. [27].

Differences were also observed in the predicted metabolic profiles of the two molecules. Xenobiotic biotransformation occurs predominantly through cytochrome P450 (CYP450) enzymes [1]. Both compounds were predicted to inhibit CYP2C9, CYP3A4, and CYP2C19 (Table S1). However, Oxβ-Lp was predicted to be a poor substrate for CYP3A4 and an inhibitor of CYP1A2, suggesting that oxime derivatization may alter its metabolic fate relative to β-lapachone. Consistent with this observation, total clearance predictions indicated a tendency toward more rapid elimination of Oxβ-Lp.

Analysis of the toxicity parameters presented in Table S2, indicated that neither compound was predicted to induce skin sensitization or inhibit hERG I and II potassium channels. This finding is particularly relevant because hERG inhibition is frequently associated with cardiac arrhythmias and drug-induced cardiotoxicity [93]. Both molecules were predicted to present hepatotoxic potential, a characteristic commonly reported for quinone-containing compounds. Although hepatotoxicity represents an important safety concern, it is also observed in several clinically used chemotherapeutic agents, including cisplatin, doxorubicin, and 5-fluorouracil [94]. In the case of β-lapachone, hepatotoxicity has been associated with hepatic redox metabolism and the generation of reactive semi-quinone intermediates [95].

Acute oral toxicity predictions suggested a slightly higher LD₅₀ value for Oxβ-Lp than for β-lapachone, indicating a potentially lower acute toxicity profile in rats. However, both compounds exhibited similar predicted chronic toxicity profiles. In addition, the lower maximum tolerated dose predicted for Oxβ-Lp highlights the importance of further experimental toxicological evaluation. Both molecules were predicted to exhibit mutagenic potential and possible carcinogenicity in mice, indicating that structural modification through oximation did not substantially alter the overall predicted toxicological profile of the parent compound.

These in silico analyses indicate that the oxime derivatization modified specific physicochemical and pharmacokinetic properties of β-lapachone, particularly water solubility, polarity, protein binding, and metabolic behavior. Although the overall toxicity profile remained broadly comparable between the two molecules, these alterations may contribute to the differences observed in the biological assays and could partially explain the distinct selectivity and cytotoxicity profiles exhibited by Oxβ-Lp. These alterations may contribute to the reduced hemolytic activity and improved selectivity observed for Oxβ-Lp relative to literature reports for β-lapachone.

### Molecular docking

Molecular docking analyses were performed to investigate the potential interaction of the naphthoquinone oxime derivative Oxβ-Lp with NAD(P)H oxidoreductase 1 (NQO1). This enzyme was selected because it plays a central role in quinone bio-activation and has been extensively implicated in the cytotoxic activity of β-lapachone and related naphthoquinones in cancer cells [28, 96]. It is important to note that docking analyses are intended to evaluate the plausibility of protein–ligand interactions and do not constitute direct evidence of enzymatic activation or substrate turnover.

The docking protocol was validated through re-docking of the native ligand dicoumarol into the NQO1 binding site (PDB ID: 2F1O). The resulting docked conformations closely overlapped with the crystallographic ligand orientation, demonstrating good reproducibility of the docking procedure (Fig. 6). Root mean square deviation (RMSD) values of 1.22 Å and 1.34 Å were obtained for the AC and BD dimers, respectively, indicating satisfactory recovery of the experimental binding pose.

The predicted binding energies for dicoumarol were − 13.47 kcal/mol and − 13.16 kcal/mol for the AC and BD dimers, respectively. Since RMSD values below 2.0 Å are generally considered indicative of a reliable docking protocol, these results support the suitability of the adopted methodology for subsequent analysis of Oxβ-Lp and β-lapachone interactions with NQO1.

**Fig. 7:**
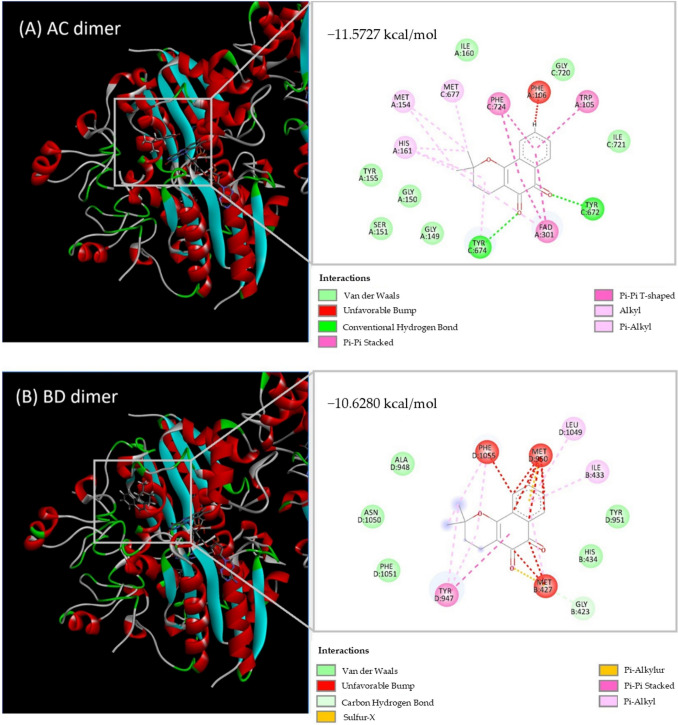
Molecular docking of the β-Lp molecule and the AC (**A**) and BD (**B**) dimers of the NQO1 enzyme

**Fig. 8:**
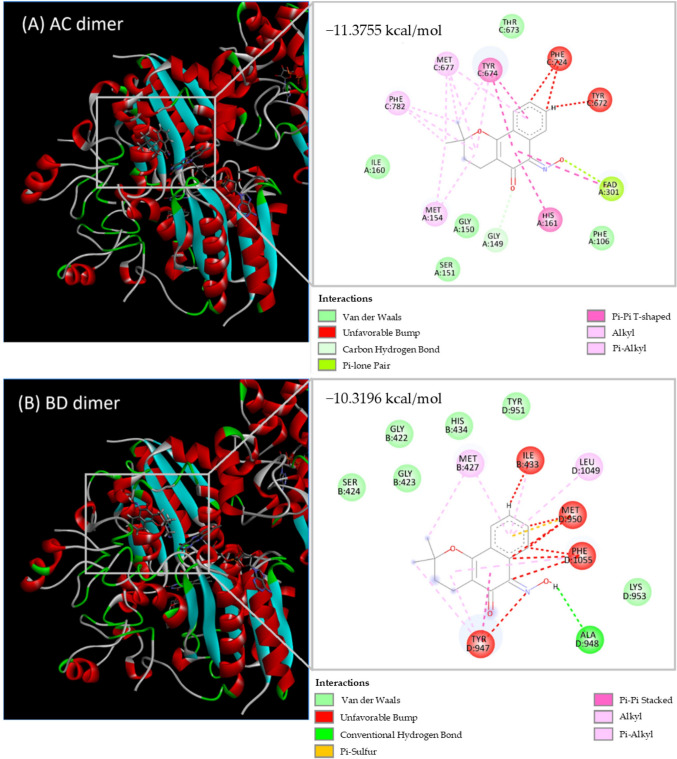
Molecular docking of the Oxβ-Lp molecule and the AC (**A**) and BD (**B**) dimers of the NQO1 enzyme

The binding poses exhibiting the highest predicted affinities for β-Lp and Oxβ-Lp within the AC and BD catalytic dimers of NQO1 were selected for interaction analysis, as shown in Figs. 7 and 8, respectively.

NQO1 is a homodimeric flavoprotein containing two catalytic sites located at the interface between its subunits. Each active site includes a flavin adenine dinucleotide (FAD) cofactor, which plays a critical role in quinone reduction and forms the structural foundation of the ligand-binding cavity [97]. Several amino acid residues have been identified as important contributors to ligand recognition and stabilization within the catalytic pocket, including Trp105, Phe106, Gly149, Gly150, Tyr155, His161, His194, Pro68, Tyr126, Tyr128, Gly174, and Phe178 [98]. Because the catalytic sites are formed by residues contributed by both monomers, ligand binding depends on a coordinated network of interactions involving the FAD cofactor and neighboring amino acid residues. Therefore, characterization of these interactions may provide valuable insights into the potential recognition of Oxβ-Lp by NQO1.

NQO1 operates through a classical ping-pong catalytic mechanism in which NAD(P)H and the quinone substrate bind sequentially to the same active site [99, 100]. During the first catalytic step, a hydride ion is transferred from NAD(P)H to the N5 atom of the flavin adenine dinucleotide (FAD) cofactor, generating reduced FADH₂. Tyr155 participates in proton transfer through hydrogen-bond interactions with FAD and is stabilized by His161. Following NAD(P)⁺ release, the quinone substrate enters the catalytic cavity and accepts a hydride from FADH₂, yielding the corresponding hydroquinone and initiating a new catalytic cycle.

The resulting hydroquinone is often unstable and may undergo spontaneous re-oxidation, generating reactive oxygen species (ROS) through redox cycling. This process has been associated with oxidative stress, DNA damage, and cell death in NQO1-expressing tumor cells [39]. Because elevated NQO1 expression has been reported in several malignancies, including melanoma, pancreatic adenocarcinoma, non-small cell lung cancer, and prostate cancer, this enzyme has emerged as an attractive target for the development of quinone-based anticancer agents [101, 102].

As shown in Fig. 7A, β-Lp exhibited its highest predicted binding affinity toward the AC catalytic site (− 11.5727 kcal/mol). The quinone carbonyl groups established hydrogen-bond interactions with Tyr126 and Tyr128, while π–π stacking interactions were observed with Trp105, Phe178, and the isoalloxazine ring of the FAD cofactor. Additional van der Waals interactions involved residues Tyr155, Ser151, Gly149, Gly150, Ile160, Phe106, and Gly174, whereas alkyl and π–alkyl interactions were observed with Met131, Met154, and His161. These interaction patterns are consistent with previous docking studies describing β-lapachone recognition within the NQO1 catalytic pocket [95].

Similar interaction profiles have been reported for other quinone substrates of NQO1. Hydrogen-bond formation with Tyr128 and Tyr126, together with π–π stacking interactions involving Trp105, Phe178, and the FAD cofactor, has been associated with enhanced substrate recognition and catalytic reduction by NQO1 [103]. Moreover, hydrogen-bond interactions involving quinone carbonyl groups may facilitate hydride transfer by modulating the electronic density of the substrate carbonyls [104].

At the BD catalytic site (Fig. 7B), the β-Lp established hydrogen-bond interactions with Gly150 and π-type interactions involving Ile160 and Leu230. Additional van der Waals contacts were observed with Ala129, Asn231, Phe232, and Gly150. In contrast to the AC site, no direct interaction with the FAD cofactor was detected, which may contribute to the lower predicted binding affinity observed for the BD dimer (− 10.63 kcal/mol).

Previous molecular modeling studies have demonstrated that efficient NQO1 substrates typically exhibit favorable donor–acceptor distances between quinone carbonyl groups and the hydride-transferring N5 atom of FAD [103, 105]. Consistent with these observations, β-lapachone displayed a binding orientation compatible with productive recognition within the NQO1 catalytic pocket, supporting previous experimental evidence demonstrating its bio-activation by this enzyme.

Oxβ-Lp exhibited interaction profiles with NQO1 that were broadly comparable to those observed for β-Lp in both the AC and BD catalytic dimers (Fig. 8). Overall, both compounds established similar types of intermolecular interactions and displayed comparable binding energies within the catalytic regions of the enzyme.

At the AC catalytic site (Fig. 8A), Oxβ-Lp presented unfavorable steric contacts with Phe178 and Tyr126. Nevertheless, favorable van der Waals interactions involving Thr127, Ile160, Gly150, Ser151, and Phe106 contributed to stabilization of the ligand within the binding pocket, resulting in a predicted binding energy of − 11.3755 kcal/mol. Like β-lapachone, Oxβ-Lp also interacted with the FAD cofactor. However, this interaction occurred through a π-lone pair, a non-covalent electrostatic interaction that may contribute to stabilization of the protein–ligand complex [106].

Within the BD catalytic site (Fig. 8B), Oxβ-Lp established a conventional hydrogen bond with Ala129, van der Waals interactions with Ser151, Gly149, Gly150, His161, Tyr132, and Lys134, and alkyl interactions with Met154 and Leu230. Although unfavorable contacts involving Ile160, Met131, Phe236, and Tyr128 were also identified, the molecule maintained a favorable predicted binding affinity (− 10.32 kcal/mol). Interestingly, the docking pose suggests accommodation within a lateral pocket adjacent to the catalytic region, formed by residues Tyr128, His194, Met154, Phe232, and Phe236 [104].

Comparison of the docking simulations revealed that both β-lapachone and Oxβ-Lp exhibited higher predicted affinities for the AC dimer than for the BD dimer, although the overall docking scores remained highly similar. These findings indicate that oxime derivatization did not substantially alter the predicted interaction pattern of the parent molecule within the NQO1 binding cavity.

Shreevatsa et al. [107] reported docking energies for several phytobioactive compounds interacting with NQO1 using ArgusLab, including dicoumarol, a well-characterized NQO1 ligand. Although direct comparison between independent docking studies should be interpreted cautiously because of methodological differences, the binding energies predicted for β-lapachone and Oxβ-Lp in the present study were within a similar range to those reported for other NQO1-interacting compounds, supporting the plausibility of their recognition by the enzyme.

Taken together, the docking results suggest that Oxβ-Lp retains structural features compatible with interaction within the NQO1 catalytic environment. However, molecular docking alone cannot establish whether Oxβ-Lp acts as a substrate, inhibitor, or activator of NQO1. Therefore, these findings should be interpreted as evidence supporting the potential recognition of Oxβ-Lp by NQO1 rather than direct proof of enzymatic bio-activation.

Although direct comparative experiments between β-lapachone and Oxβ-Lp were not performed in the present study, literature data suggest that oxime derivatization may alter physicochemical properties, selectivity, and toxicity profiles. Future comparative studies under identical experimental conditions will be important to clarify the pharmacological advantages and limitations associated with this structural modification.

A limitation of the present study is the absence of direct experimental validation of NQO1 involvement in Oxβ-Lp activity. Although molecular docking suggested a potential interaction with this enzyme, NQO1 expression, enzymatic activity, pharmacological inhibition, and target engagement were not evaluated. Consequently, any association between NQO1 status and Oxβ-Lp sensitivity should be considered exploratory and hypothesis-generating. Future studies incorporating NQO1 inhibition assays, gene expression analyses, and protein-level investigations will be necessary to clarify the contribution of this enzyme to the biological activity of Oxβ-Lp.

## Conclusion

Oxβ-Lp demonstrated selective cytotoxic activity against NCI-H460 non-small cell lung cancer cells and induced apoptosis associated with mitochondrial dysfunction and DNA damage. The compound also exhibited genotoxic activity in complementary experimental models and showed favorable selectivity relative to non-tumor cells. Although molecular docking suggested a potential interaction with NQO1, the involvement of this enzyme was not experimentally investigated and therefore remains to be established. Collectively, these findings support Oxβ-Lp as a promising lead compound for further anticancer development and warrant additional studies to elucidate its molecular mechanism of action and therapeutic potential.

## Supplementary Information

Below is the link to the electronic supplementary material.

Supplementary file1 (DOCX 632 KB)

## Data Availability

All data generated or analyzed during this study are included in the article.
