# Supplementary material for: Cytotoxic and genotoxic effects of oxime β-lapachone in human cancer cells: selectivity toward NCI-H460 and insights from molecular docking
Source: Hum Cell. 2026 Jul 27;39(8):117. doi: 10.1007/s13577-026-01427-8 (PMC13408113; doi:10.1007/s13577-026-01427-8)
Supplement: Supplementary file 1 — Supplementary file1 (DOCX 632 KB) [file 13577_2026_1427_MOESM1_ESM.docx]

**SUPPLEMENTARY DATA**

**Cytotoxic and Genotoxic Effects of Oxime β-Lapachone in Human Cancer Cells: Selectivity Toward NCI-H460 and Insights from Molecular Docking**

**Table of contents**

**Figure S1.** Chromosomal alterations in meristematic cells of Allim cepa roots after 48 h of exposure to Oxβ-Lp at a concentration of 7.78 μM (A, B, C: magnification ×1000; D: magnification ×400). (A and B) Chromosomal fragments (black arrows); (C) Delay (black arrow) and chromosomal fragments (white arrow); (D) Chromosomal bridge (black arrow).

**Figure S2.** Computational predictions panel of the Oxβ-Lp molecule, including parameters such as chemical structure, bioavailability Radar, physicochemical properties, lipophilicity, water solubility, pharmacokinetics, druglikeness, and medicinal chemistry.

**Figure S3.** Computational predictions panel of the β-Lp molecule, including parameters such as chemical structure, bioavailability Radar, physicochemical properties, lipophilicity, water solubility, pharmacokinetics, druglikeness, and medicinal chemistry.

**Figure S4.** The BOILED-Egg plot demonstrates that both molecules have human intestinal absorption (HIA) (white ovule) and can cross the blood-brain barrier (BBB) (yellow ovule). The red dots indicate that neither molecule can act as a substrate for P-glycoprotein (P-gp).

**Table S1.** Pharmacokinetic profile of Oxβ-Lapachone (Oxβ-Lp) and β-Lapachone (β-Lp).

**Table S2.** Toxicity predictions of Oxβ-Lapachone (Oxβ-Lp) and β-Lapachone (β-Lp).


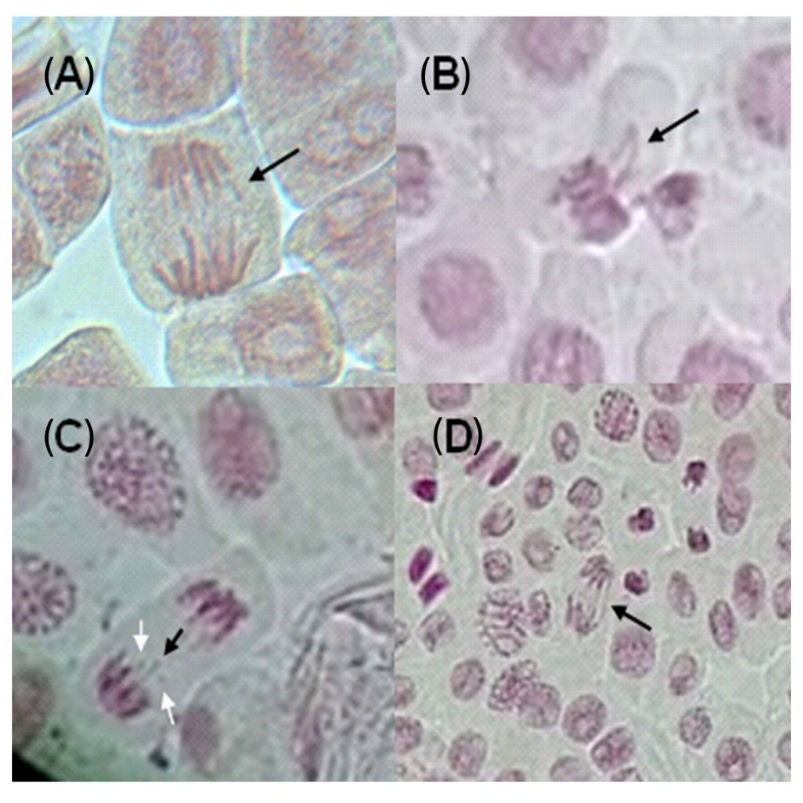


**Figure S1**. Chromosomal alterations in meristematic cells of Allim cepa roots after 48 h of exposure to Oxβ-Lp at a concentration of 7.78 μM (A, B, C: magnification ×1000; D: magnification ×400). (A and B) Chromosomal fragments (black arrows); (C) Delay (black arrow) and chromosomal fragments (white arrow); (D) Chromosomal bridge (black arrow).

**
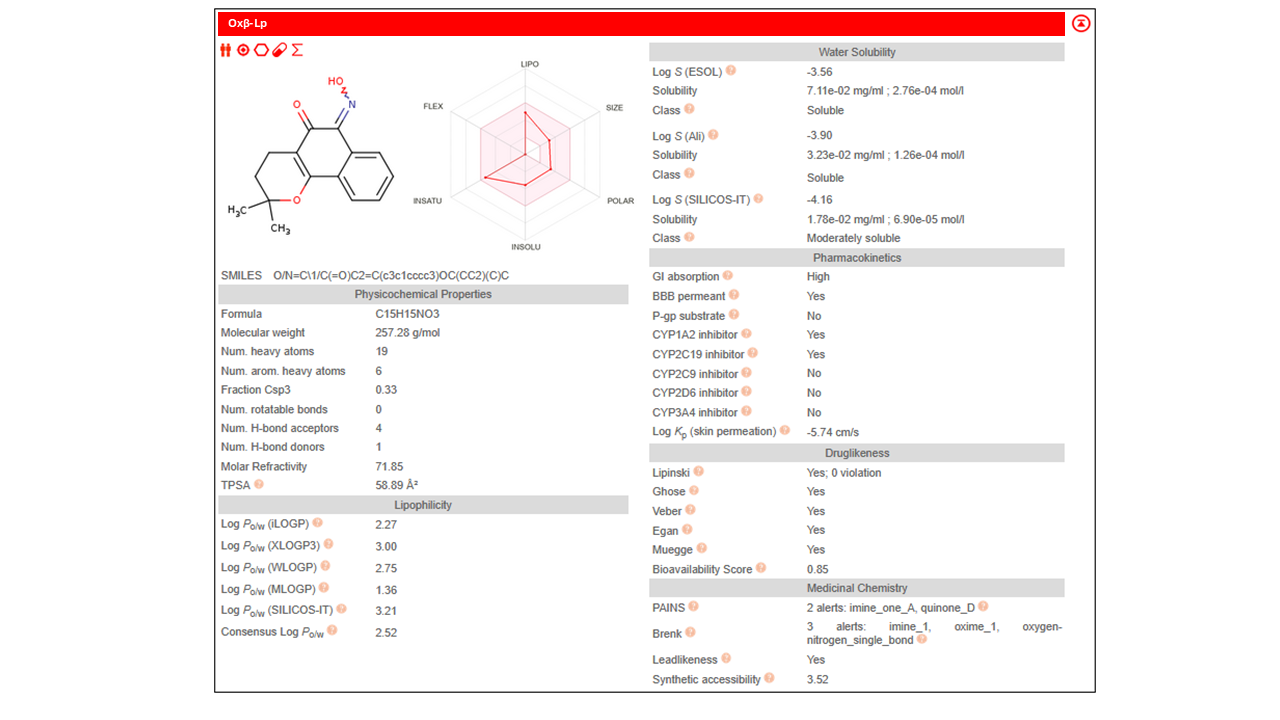
**

**Figure S2.** Computational predictions panel of the Oxβ-Lp molecule, including parameters such as chemical structure, bioavailability Radar, physicochemical properties, lipophilicity, water solubility, pharmacokinetics, druglikeness, and medicinal chemistry.

**
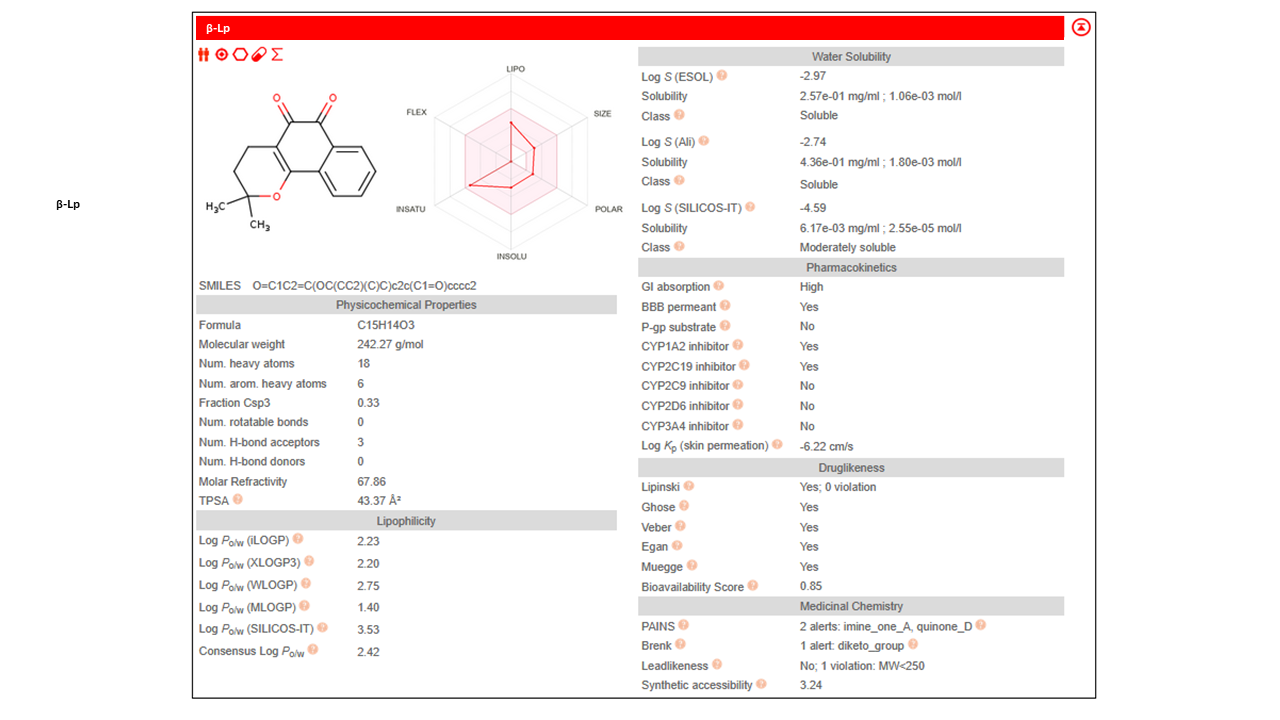
**

**Figure S3.** Computational predictions panel of the β-Lp molecule, including parameters such as chemical structure, bioavailability Radar, physicochemical properties, lipophilicity, water solubility, pharmacokinetics, druglikeness, and medicinal chemistry.

**
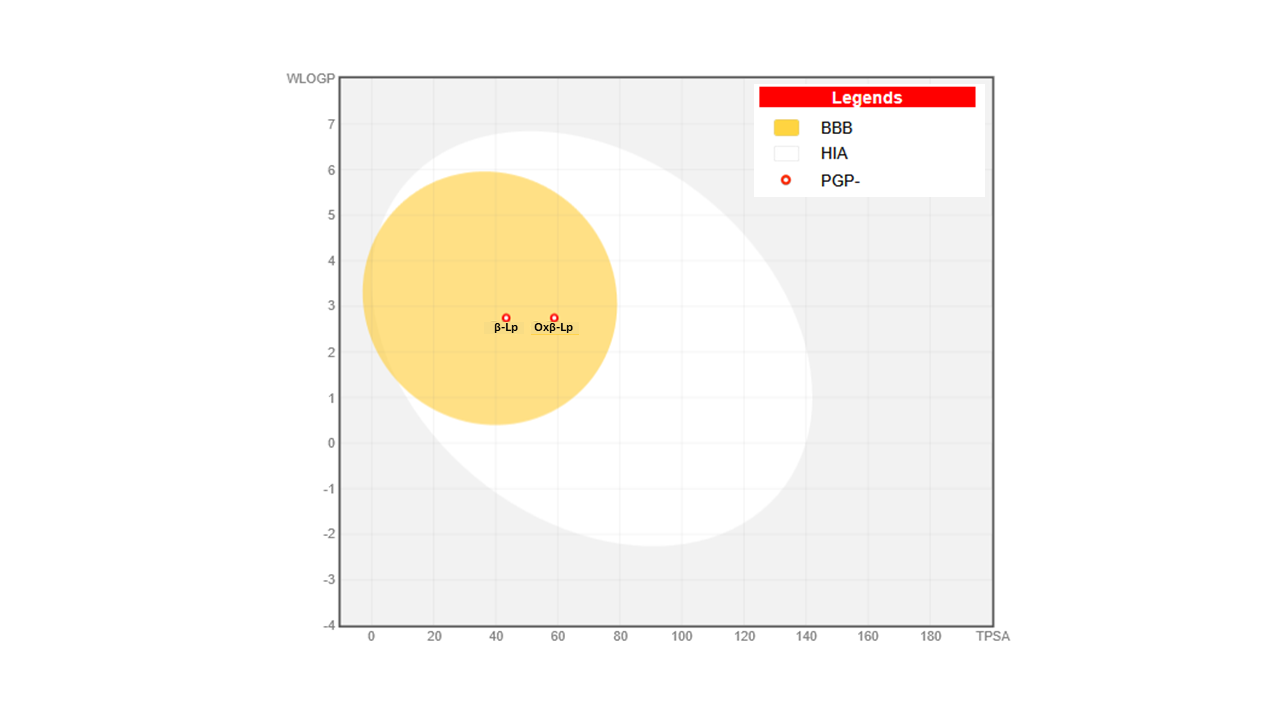
**

**Figure S4.** The BOILED-Egg plot demonstrates that both molecules have human intestinal absorption (HIA) (white ovule) and can cross the blood-brain barrier (BBB) (yellow ovule). The red dots indicate that neither molecule can act as a substrate for P-glycoprotein (P-gp).

**Table S1.** Pharmacokinetic profile of Oxβ-Lapachone (Oxβ-Lp) and β-Lapachone (β-Lp).

|  | **Profile** | | | **Oxβ-Lp** |  | | **β-Lp** |  |
| --- | --- | --- | --- | --- | --- | --- | --- | --- |
| **Absortion** | ^a^Free energy of solvation | | | -7.50 |  | | -9.35 |  |
|  | ^a^Human intestinal absorption (%) | | | 95.39 |  | | 97.94 |  |
|  | ^a^MDCK permeability (nm/s) | | | 105.49 |  | | 223.40 |  |
|  | ^b^P-glycoprotein substrate/inhibitor | | | No |  | | No |  |
| **Distribution** | ^a^Plasma protein binding (%) | | | 85.90 |  | | 98.28 |  |
|  | ^b^BBB permeability (log BB) | | | 0.173 |  | | 0.105 |  |
| **Metabolism** | ^a^CYP3A4 substrate |  | Weakly | | | Yes | | |
|  | ^a^CYP3A4 inhibitor |  | Yes | | | Yes | | |
|  | ^a^CYP2D6 substrate |  | No | | | No | | |
|  | ^a^CYP2D6 inhibitor |  | No | | | No | | |
|  | ^a^CYP2C9 inhibitor |  | Yes | | | Yes | | |
|  | ^a^CYP2C19 inhibitor |  | Yes | | | Yes | | |
|  | ^a^CYP1A2 inhibitor |  | No | | | Yes | | |
| **Excretion** | ^b^ Total clearance (log mL/min/kg) |  | 0.192 | | | 0.102 | | |
|  | ^b^OCT2 substrate |  | No | | | No | | |

^a^PreADMET; ^b^pkCSM; MDCK: Madin-Darby canine kidney; BHE: blood-brain barrier; OCT2: organic cation transporter 2.

**Table S2.** Toxicity predictions of Oxβ-Lapachone (Oxβ-Lp) and β-Lapachone (β-Lp).

|  |  | Oxβ-Lp | β-Lp |
| --- | --- | --- | --- |
| Toxicity | ^b^Maximun tolerated dose (human) (log mg/kg/day) | 0.416 | 0.543 |
|  | ^b^hERG I/II inhibitor | No | No |
|  | ^b^Oral rat acute toxicity (LD_50_) (mol/kg) | 2.263 | 1.946 |
|  | ^b^Oral rat chronic toxicity (LD_50_) (mg/kg/day) | 1.915 | 1.963 |
|  | ^b^Hepatotoxicity | Yes | Yes |
|  | ^b^Skin sensitization | No | No |
| Mutagenicity | ^a^Ames test | Mutagenic | Mutagenic |
| Carcinogenicity | ^a^Rat | Negative | Negative |
|  | ^a^Mice | Positive | Positive |

^a^PreADMET; ^b^pkCSM; hERG (*Human Ether-a-go-go Related Gene Potassium Channel*); LD_50_: Lethal Dose 50%.
